# Electrophysiological Signatures of Numerosity Encoding in a Delayed Match-to-Sample Task

**DOI:** 10.3389/fnhum.2021.750582

**Published:** 2022-01-04

**Authors:** Wanlu Fu, Serena Dolfi, Gisella Decarli, Chiara Spironelli, Marco Zorzi

**Affiliations:** ^1^Department of General Psychology, University of Padova, Padua, Italy; ^2^Department of Developmental Psychology and Socialisation, University of Padova, Padua, Italy; ^3^Padova Neuroscience Center (PNC), University of Padova, Padua, Italy; ^4^IRCCS San Camillo Hospital, Venice, Italy

**Keywords:** approximate number system, subitizing, symbolic numbers, mathematics, numerosity encoding, event-related potentials

## Abstract

The number of elements in a small set of items is appraised in a fast and exact manner, a phenomenon called subitizing. In contrast, humans provide imprecise responses when comparing larger numerosities, with decreasing precision as the number of elements increases. Estimation is thought to rely on a dedicated system for the approximate representation of numerosity. While previous behavioral and neuroimaging studies associate subitizing to a domain-general system related to object tracking and identification, the nature of small numerosity processing is still debated. We investigated the neural processing of numerosity across subitizing and estimation ranges by examining electrophysiological activity during the memory retention period in a delayed numerical match-to-sample task. We also assessed potential differences in the neural signature of numerical magnitude in a fully non-symbolic or cross-format comparison. In line with behavioral performance, we observed modulation of parietal-occipital neural activity as a function of numerosity that differed in two ranges, with distinctive neural signatures of small numerosities showing clear similarities with those observed in visuospatial working memory tasks. We also found differences in neural activity related to numerical information in anticipation of single vs. cross-format comparison, suggesting a top-down modulation of numerical processing. Finally, behavioral results revealed enhanced performance in the mixed-format conditions and a significant correlation between task performance and symbolic mathematical skills. Overall, we provide evidence for distinct mechanisms related to small and large numerosity and differences in numerical encoding based on task demands.

## Introduction

Humans possess the ability to rapidly assess the number of items in a set (numerosity) without the necessity to count the objects. However, the speed and precision of these numerical judgments show different patterns depending on the number of elements to be estimated. Individuals provide fast and errorless responses in the case of sets composed of a few items (up to four), a phenomenon named subitizing (Kaufman and Lord, 1949). Instead, estimates of larger sets tend to be imprecise, with variability increasing proportionally to the number of objects (Jevons, 1871).

This dichotomy led to the hypothesis that subitizing and estimation are subserved, at least partially, by different mechanisms (see Piazza, 2010 for review). Numerosity estimation is thought to rely on the Approximate Number System (ANS; Feigenson et al., 2004), a preverbal mechanism characterized by the noisy encoding of numerical information (Feigenson et al., 2004). The ANS is often modeled as a mental number line where numerical magnitudes are coded as Gaussian distributions showing an increase in overlap as numerosity increases (either due to scalar variability or compressive scaling; Gallistel and Gelman, 1992; Dehaene, 2003), thereby accounting for the imprecise estimation of large sets and ratio-dependent performance in comparing different numerosities (in accordance with Weber’s law). The same pattern of performance is also shown by state-of-the-art computational models of numerosity perception based on deep neural networks (Stoianov and Zorzi, 2012; Zorzi and Testolin, 2018; Nasr et al., 2019; Testolin et al., 2020). However, it has been proposed that numerosity mechanisms may work only at low to moderate densities, where items can be segregated. At higher densities, where objects become crowded, texture-like mechanisms may operate (Anobile et al., 2014, 2015). Moreover, performance in the subitizing range violates Weber’s law, so that accuracy and reaction times in numerical comparison or same-different tasks remain stable across the entire range (Choo and Franconeri, 2014). Accordingly, the subitizing phenomenon has been related to a domain-general system for object identification and localization in space, named Object Tracking System (OTS) (Trick and Pylyshyn, 1994).

Nonetheless, the nature of subitizing remains contentious. An alternative view proposes that subitizing effects are a by-product of the scalar variability of the numerical representation, which would equally predict a high level of precision for smaller numerosities (Gallistel and Gelman, 1992). However, participants are faster and more precise in comparing pairs of numerosities in the subitizing range with respect to pairs in the estimation range with a similar ratio (Revkin et al., 2008). Nevertheless, two recent computational studies have revealed that distinct patterns of behavioral performance in subitizing and estimation range could potentially emerge from a single flexible system (Sengupta et al., 2017; Cheyette and Piantadosi, 2020), reinvigorating the debate.

In support of the idea of separate systems, different developmental trajectories have been described for subitizing and estimation abilities. The limited capacity of OTS improves during the first year of life, from a range of 1–2 items, up to the adult-like average limit of four objects (Coubart et al., 2014). In contrast, the precision of ANS seems to increase more steadily over the entire lifespan (Halberda et al., 2012). In addition, considerable individual differences exist both in the limit of objects that can be subitized and in the precision of large numerosity discrimination (Halberda et al., 2008; Piazza et al., 2011). However, individual subitizing limits seem not to be correlated with estimation precision (Piazza et al., 2011). Notably, converging evidence suggests a connection between individual differences in estimation precision (also known as number acuity) and more advanced mathematical skills, at least in developmental populations (Halberda et al., 2008; Mazzocco et al., 2011b; Starr et al., 2013; Chen and Li, 2014; van Marle et al., 2014). A widely accepted interpretation of this link is that the ANS may play a scaffolding role in the acquisition of symbolic numerical knowledge (Piazza et al., 2010; but see Leibovich and Ansari, 2016). In this view, during the acquisition of counting, individuals would create a mapping of symbolic numerals (Arabic digits or number words) onto the preexisting analog representations of numerical magnitudes (Gallistel and Gelman, 1992). Indeed, size and distance effects have also been reported in the case of comparison of symbolic numerals (e.g., Mussolin et al., 2010). It must, however, be noted that some authors have argued in favor of fully distinct processing for symbolic numbers, claiming that psychophysical similarities with ANS are limited to tasks where numerosities and numerals are interleaved, and observing a cognitive cost when symbolic and non-symbolic information needs to be integrated (Lyons et al., 2012; Sasanguie et al., 2017; Marinova et al., 2020). Indeed, although mathematical competence has been reliably related to both non-symbolic and symbolic magnitude processing (De Smedt et al., 2013), a stronger association with symbolic comparison abilities has been reported, especially in adults (Castronovo and Göbel, 2012; Schneider et al., 2017).

In contrast, individual variability in subitizing capacity has not been reliably associated with arithmetic skills (Anobile et al., 2019). Moreover, individuals with specific difficulties in mathematics (developmental dyscalculia) show impaired numerosity estimation (Piazza et al., 2010; Mazzocco et al., 2011a) but intact subitizing capacity (Decarli et al., 2020; but see Schleifer and Landerl, 2011). Conversely, impaired subitizing (but not estimation) has been observed in individuals with Down syndrome (Sella et al., 2013), who are also known to suffer from visuospatial working memory deficits. Accordingly, subitizing has been linked to domain-general visuospatial processing mechanisms. In particular, subitizing requires attentional resources and is disrupted by dual tasks with high attentional demands (Piazza et al., 2011), whereas numerosity estimation is carried out by a pre-attentive mechanism and is minimally affected by attentional load (Burr et al., 2010). Finally, individual subitizing limits have been related to visual working memory capacity (Piazza et al., 2011) and can be improved by cognitive training involving visuospatial abilities (Green and Bavelier, 2003).

Neuroimaging studies have consistently associated numerical processing with frontoparietal cortical circuits (Piazza et al., 2006). More specifically, fMRI activity in the bilateral intraparietal sulcus (IPS) elicited by deviant stimuli in a number adaptation paradigm has shown ratio dependency, a signature effect of ANS (Piazza et al., 2004). Moreover, this area has been related to magnitude processing during numerical judgments (e.g., Eger et al., 2015) and approximate computation (e.g., Bugden et al., 2019), although recent studies revealed that separate subregions near the IPS could be differentially engaged during different tasks (Castaldi et al., 2020). Moreover, in line with the idea of a partially shared semantic representation, neural responses to Arabic digits and number words were individuated in areas associated with magnitude processing, although additional lateralized circuits seem to be implicated in symbolic representation (Eger et al., 2003; Piazza and Eger, 2016; Sokolowski et al., 2017; but see Bulthé et al., 2014). Several studies report that bilateral IPS activation is sensitive to numerical magnitude changes in response to both symbolic and non-symbolic stimuli or cross-format presentation, with stronger effects on the left IPS in the case of symbolic stimuli (Piazza et al., 2007; Notebaert et al., 2011). Moreover, TMS studies showed that performance in non-symbolic comparison could be disrupted by bilateral parietal stimulation, while only left stimulation on similar sites was sufficient to impair digit discrimination (Andres et al., 2005; Cappelletti et al., 2007). In sum, these results relate the left hemisphere with the processing of exact numerical information and more refined coding of numerical magnitude, possibly because of connections with frontal circuits involved in language processing (Ansari, 2007). This idea is also consistent with a progressive left shift in the lateralization of number-related activity during development, which suggests an increasing differentiation of the symbolic representation from the magnitude system as formal mathematical concepts are learned (Emerson and Cantlon, 2015).

Only a few neuroimaging studies have examined both subitizing and estimation within the same experimental paradigm. Notably, in an fNIRS study, Cutini et al. (2014) found dissimilarities in the hemodynamic response of IPS to small and large numerosities, revealing a non-linear increase with numerosity in response amplitude. Also, a specific implication of the temporoparietal junction (TPJ) during small compared to large number discriminations supports the idea that a separate system could intervene in the processing of small numerosities (Ansari et al., 2007).

Importantly, previous electrophysiological studies based on the event-related potentials (ERPs) have produced mixed results. Although modulation of activity in posterior parietal sites has been reported both in response to large and small sets of objects, the use of different paradigms, procedures, and stimulus formats is the likely cause of discrepancies in timing and polarity of numerosity-related effects across studies. A positive component around 200 ms after stimulus onset has been found to increase in amplitude for small distances or ratios in symbolic and non-symbolic comparison tasks (Dehaene, 1996; Temple and Posner, 1998; Turconi et al., 2004) or passive viewing (Hyde and Spelke, 2009; Liu et al., 2018). While these effects are often interpreted as signatures of approximate magnitude processing, it must be noted that many of these studies mixed sets or numerals from the estimation and subitizing range. Moreover, modulation by numerical ratio with opposite polarity was found in a similar time window by Rubinsten et al. (2013). Using a non-symbolic match-to-sample task, instead, other authors found a distance effect in later negative deflections (300–500 ms), with a larger amplitude for close compared to far distances (Paulsen and Neville, 2008; Paulsen et al., 2010), while others failed to find distance effects even for early ERP components (van Hoogmoed and Kroesbergen, 2018).

In contrast, an earlier modulation (around 150 ms) has been found in response to small non-symbolic numbers. In this case, the amplitude is more reliably reported to increase as a function of the absolute magnitude for small, but not for large, numerosities (Libertus et al., 2007; Hyde and Spelke, 2009, 2012; Fornaciai and Park, 2017) or for mismatch compared to match conditions (Liu et al., 2018). However, some studies also report modulation by distance in this time window (Temple and Posner, 1998; Merkley et al., 2016). Moreover, Park et al. (2016) recently demonstrated that monotonic modulation of parietal activity by numerosity can also be appreciated for larger numerosities in positive peaks around 220 ms. Another line of research focusing on the link between small numerosity processing and attentional functions revealed that neural signatures of object individuation (e.g., N2pc) are modulated by target numerosity up to a fixed limit and correlated with individual subitizing span (Ester et al., 2012; Mazza et al., 2013).

In sum, previous ERP studies on number processing have examined magnitude effects during passive viewing or distance/ratio effects elicited by the comparison of pairs of stimuli, but the results are inconclusive regarding the distinction between small and large numerosities, and their putative relationship with different neurocognitive systems (OTS vs. ANS). In contrast, research on object individuation and/or visual working memory (WM) offers a potential alternative perspective for the investigation of numerosity encoding. More specifically, ERP signatures of memory retention (e.g., contralateral delay activity, CDA) during spatial or object working memory tasks have been reliably shown to be modulated by set size (McCollough et al., 2007; Drew et al., 2012). For example, during the retention period of a memory display in a change detection task, a negative slow wave in parietal sites increased in amplitude, as a function of the number of objects to be remembered, but only by up to 4–6 items (Feldmann-Wüstefeld, 2021). Recently, using an enumeration paradigm with a delay period between stimulus presentation and response prompt, Pagano et al. (2014) observed a similar modulation of CDA by the number of items to be enumerated, thereby showing involvement of working memory processes in subitizing during an explicitly numerical task. A similar approach has been previously used in numerical research in comparative studies investigating monkeys’ neuronal response to numerosity during the delay period of a match-to-sample task. These studies revealed the presence of neurons maximally activated by specific numerosities in the prefrontal and posterior parietal cortices (Nieder and Miller, 2004; Nieder et al., 2006; Tudusciuc and Nieder, 2007; Viswanathan and Nieder, 2013). Taken together, these findings suggest that examining neural activity during encoding and maintenance of numerical information could help elucidate possible differential processing of small and large numerosities.

In this study, we exploited a delayed numerosity match-to-sample task (Sella et al., 2013) in which participants had to report if the numerosity of a dot array (sample stimulus) matched (or mismatched) the numerosity of a subsequent test stimulus presented after a 1-s blank-display delay period. More specifically, we hypothesized that EEG activity between sample stimulus offset and second test stimulus onset (i.e., the memory period) would be influenced by the number of items in the array, as typically observed in single-cell neurophysiological studies (e.g., Nieder and Miller, 2004). Importantly, we investigated neural activity during the delay period to avoid confounds from comparison processes and response-related activity. The memory-related activity is also less likely to be affected by changes in task-irrelevant perceptual attributes of the sample stimulus, such as individual dot size, cumulative area, etc., since variability linked to visual processing is usually found in early ERP components (e.g., P1, N270) and generally within 300 ms from stimulus onset (Luck, 2014; Soltész and Szucs, 2014; Park et al., 2016). Crucially, to investigate potential differences in the encoding of small and large numerosities, the number of items in the arrays spanned from subitizing to estimation range. Moreover, at an exploratory level, we investigated whether matching sample numerosity with an Arabic digit (i.e., a cross-format match-to-sample condition) would lead to a more distinctive neural signature of numerical magnitude. From behavioral evidence reporting differences in the comparison of within-format or multi-format numerical information (Marinova et al., 2020) and neuroscientific support in favor of format-dependent neural representation of numerosity (Eger et al., 2009), we hypothesized that the cross-format presentation, because of the symbolic nature of the test stimulus, could induce more precise encoding of the sample numerosity, irrespective of range. This “dots-to-digit” condition was presented as a separate block, but the sample stimulus remained identical to that of the “dots-to-dots” condition. Therefore, our focus remained on ERPs elicited by the non-symbolic sample stimulus to examine differences in physically equal stimuli varying only in psychological conditions (i.e., the format of the test stimulus), according to Hillyard’s principle. We also asked if signature patterns of subitizing and estimation would be present at the behavioral level and whether the performance would differ in the two conditions. Finally, we offer our contribution to the ongoing debate on the relationship between basic numerical abilities and broad mathematical skills by looking at the correlation between behavioral performance in the match-to-sample task and more advanced arithmetic abilities assessed during the study.

## Materials and Methods

### Participants

Thirty participants took part in the study, after giving written informed consent. Twelve of them received a monetary reward for participating. All the participants had a normal or corrected-to-normal vision. Data from two of the participants were discarded because of poor understanding of task instructions and low performance in the easiest task conditions (see below). The final sample, thus, consisted of twenty-eight participants (18 women, age range: 18–29 years). The sample size was set to be larger than the average of previous ERP studies (i.e., 20 participants) investigating symbolic or non-symbolic number comparison that reported reliable waveform modulations across numerical conditions (Libertus et al., 2007; Heine et al., 2013; Pagano et al., 2014; Fornaciai and Park, 2017). The research procedures were approved by the Psychological Science Ethics Committee of the University of Padova.

### Tasks and Stimuli

#### Match-to-Sample Task

**FIGURE 1:**
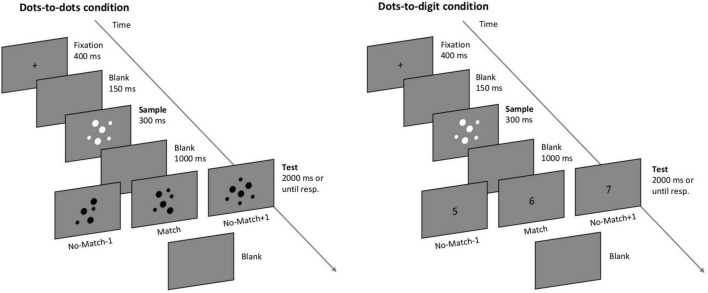
Depiction of the match-to-sample paradigm. The participants were asked to decide if the numerosity of the second test stimulus matched the numerosity of a sample dot array. In the dots-to-dots condition, the test stimulus consisted of a dot-array, while in the dots-to-digit condition the test stimulus was an Arabic digit.

All the participants performed a computerized delayed numerosity match-to-sample task (Sella et al., 2013) divided into two blocks corresponding to different task conditions (see Figure 1). In the first block (dots-to-dots condition), each trial was composed of two sequentially presented images of dot arrays, and the participants had to report if the two images contained the same number of dots. In the second block (dots-to-digit condition), the second stimulus was an Arabic digit, and the participants were asked to report if the number of dots of the first stimulus matched the number indicated by the digit. Each trial started with a fixation cross appearing in the center of the screen for 400 ms, followed by a blank screen lasting for 150 ms. Then, the first stimulus (sample, always an array of dots) was displayed centrally for 300 ms, followed by another blank interval of 1 s (delay period). The second test stimulus was presented at the center of the screen for a maximum of 2 s, after which a blank screen was presented until response. Participants pressed the left key of the mouse to indicate a matching pair or the right key to indicate an unequal number of dots in the two images or a mismatching digit. The next trial started as soon as a response was provided. Each block consisted of 130 trials, with a short break after 40 and 80 trials. The participants performed six practice trials before the dots-to-dots block and four practice trials before the dots-to-digit block. All the practice trials were identical to the respective test block. The order of the two blocks was kept constant: the aim was to initially engage the participants in a fully non-symbolic condition before the cross-format condition, as the latter might trigger symbolic (e.g., verbal) coding of the sample numerosity in order to match it with the upcoming digit.

The numerosity of the stimuli ranged from 1 to 8. In mismatching trials, the test stimulus could differ from the sample by one less or one more, with equal probability. For example, a sample numerosity of 2 could be followed by a 2 (match) or a 1 or a 3 (mismatch). Each sample numerosity was presented 10 times in a matching pair and 10 times in a non-matching pair for each block, with the exception of numerosities 1 and 8, which were only compared with 2 and 7, respectively, always in a mismatching pair. Therefore, the six match pairings were 2 vs. 2, 3 vs. 3, 4 vs. 4, 5 vs. 5, 6 vs. 6, and 7 vs. 7, while the 7 mismatching pairings were 1 vs. 2, 2 vs. 3, 3 vs. 4, 4 vs. 5, 5 vs. 6, 6 vs. 7, and 7 vs. 8 (including the opposite combinations). On each trial, a pair was randomly selected from the entire list of possible pairings in order to minimize participants’ expectations on the upcoming stimulus.

Dot arrays were created online during the experiment. The size and spatial arrangement of each dot in a grid were selected randomly on each trial. In the dots-to-dots condition, an opposite contrast polarity was used to present sample and test arrays, respectively, with white and black dots on a gray background. In the dots-to-digit condition, sample dot arrays were presented in white, while Arabic digits were presented in black as bold Courier New text, size 30. The individual area of the dots and contrast polarity were varied to minimize the influence of visual characteristics on participants’ performance. The experimental task was presented with E-Prime 2 (Psychology Software Tools, 92 Pittsburgh, PA) on a 19′ monitor screen with a resolution of 1,024 × 768, running at 60 Hz.

#### Calculation Skills

All the participants were also administered two calculation tests taken from a standardized battery for the assessment of literacy and numeracy skills in adults (LSC-SUA) (Montesano et al., 2020). This battery is used in Italy to evaluate learning disabilities in university students and adults. Participants performed two subtests: Mental Calculation and Approximate Calculation.

The Mental Calculation (MC) test consists of ten orally presented arithmetic operations (three additions, two subtractions, three multiplications, and two divisions) to be solved as rapidly and accurately as possible. In each computation, one of the numbers was a two- or three-digit number. The participants were asked to verbally report the result for each operation within 30 s. The experimenter recorded the number of correct responses and the time taken for each calculation.

The Approximate Calculation (AC) test consists of sixteen difficult arithmetic operations, presented to participants as written multiple-choice questions. The participants were asked to indicate for each operation the correct answer among three alternatives, trying to answer as many questions as they could within 1 min. The participants were explicitly instructed to avoid precise calculation to prioritize the speed of response.

### Procedure

The experiment was conducted in a quiet room, in a single session lasting approximately 1 h. Each participant first performed the paper-and-pencil test involving calculation skills. Then, after a brief resting period, they performed the numerical task while electroencephalography (EEG) was being recorded. The participants also completed a reading test at the beginning of the session and two additional computerized tasks tapping into phonological and visuospatial skills at the end of the session. These additional measures are not relevant to this study and will not be considered here.

### Electrophysiological Recording

An elastic cap (actiCAP; Brain Products, Gilching, Germany) with 64 pre-amplified electrodes mounted according to the International 10–20 system (Oostenveld and Praamstra, 2001) was used. Data were stored using the Brain Vision Recorder (Brain Products GmbH, Germany) system. The sampling rate was set at 1,000 Hz, and impedance was kept below 5 kΩ. All cortical electrodes were online referred to FCz electrodes.

### Data Analysis

#### Analysis of Behavioral Performance

In the delayed match-to-sample task, we excluded outlier trials where the response was recorded before 200 ms (anticipation) or later than 2 s (maximum image display time). With this procedure, we discarded a total of 122 trials in the entire sample (∼1.6%). The analysis focused on test trials with sample or test numerosity from 1 to 7, excluding the maximum numerosity presented (8) to avoid guessing-end effects (Simon et al., 1998). We also inspected individual performance to ensure that the participants were correctly engaged in the task, which resulted in the exclusion of two participants. One was excluded because of the accuracy level in the easiest condition (1 vs. 2 or 2 vs. 1) that did not differ from chance according to a binomial test. The other was excluded because of an unusual pattern of responses, with lower accuracy in the small numerosity range than in larger numerosities. The data analysis focused on the proportion of correct responses and mean response times (RTs) in correct trials.

To analyze the effect of numerosity on performance, we first computed mean accuracy and RTs for each participant, condition, and sample numerosity. A preliminary ANOVA with numerosity and condition as within subject levels was performed to assess the overall effect of sample numerosity and its interaction with the condition. To investigate potential differences in performance in response to small and large numerosities, we first estimated the subitizing threshold at group level, separately for each condition, by fitting a piecewise linear model to describe group mean error rates as a function of sample numerosity (Pagano et al., 2014). The inflection point of the bilinear model in the dots-to-dots condition was 3.55 (*R*^2^_adj_ = 0.9), while the breakpoint estimated on dots-to-digit trials was 5.33 (*R*^2^_adj_ = 0.97). Note that similar estimates were derived when group subitizing thresholds were estimated with the method of Leibovich-Raveh et al. (2018), which returned thresholds of 4.07 and 5.18 for the dots-to-dots and dots-to-digit conditions, respectively. Based on the bilinear thresholds, mean accuracy and mean RTs were then separately computed for each participant and condition, across trials in small (pre-inflection) and large (post-inflection) numerical ranges. Differences between conditions and numerical ranges were then investigated by repeated measures ANOVAs.

To better investigate the pattern of responses to large and small numerosities, we also applied a bilinear fit on individual mean error rates as a function of numerosity, estimating individual breakpoints and pre-and post- inflection slopes separately for each participant and condition. During this procedure, one participant was excluded because of a lack of variability in their response. Mean model fit across conditions was *R*^2^_adj_ = 0.57. Slopes across ranges and conditions were compared by means of repeated measure ANOVAs, while breakpoints in the two conditions were compared by a paired-sample *t*-test. A comparison between pre- and post-inflection slopes was also carried out on response times, fitting for each participant and condition a segmented model with a fixed breakpoint equal to the corresponding inflection point based on the error rate function. In all the analyses, Greenhouse-Geisser correction was applied in case of violation of the sphericity assumption, and *post hoc* tests were corrected for multiple comparisons with the Bonferroni method.

A correlation analysis was also performed to investigate the relationship between performance in the match-to-sample task and formal arithmetic skills. Standardized scores were computed for both subtests according to the Italian normative data of the LSC-SUA. Performance in the match-to-sample task was summarized for each condition using balanced integration scores (BISs), computed for each participant as the difference between the proportion of correct responses and mean correct response times, both standardized across conditions and participants (Liesefeld and Janczyk, 2019; Vandierendonck, 2021). Pearson correlations, controlled for FDR at alpha equal to 0.05, were then computed between BISs in the task and correctness scores from MC and AC, and the timing score from the MC test.

The data were analyzed with R (package SiZer), MATLAB (R2020a), and JASP (ver. 0.12.1 2020).

#### EEG Analysis

After data collection, all cortical electrodes were re-referenced off-line to the mean activity of the whole scalp by the average reference procedure. Signal analysis was then carried out using the Brainstorm toolbox (Tadel et al., 2011). After a 0.1–45 Hz band-pass filter, eye movement artifact components (i.e., blinking, vertical, and horizontal movements) were corrected by applying Independent Component Analysis (ICA) transformation to the EEG signal. Since, as mentioned in the procedure, we analyzed the cortical activity elicited by sample stimuli, and numerosities 1 and 8 had less trial numbers than numerosities 2–7, the following ERP analysis excluded numerosities 1 and 8. Signals for numerosities 2–7 were then segmented into 1,050-ms epochs, ranging between 200 ms before the onset of sample stimuli and 850 ms after stimulus presentation. A baseline correction (−200 to 0 ms) was applied to all the epochs. After a second 0.5–30 Hz band-pass filtering, epochs with amplitude exceeding ± 75 μV were rejected. With this artifact rejection procedure, around 2.11 trials (10.55%) were rejected in each minimum experimental cell for each participant. Another baseline correction (−200 to 0 ms) was performed right before the grand average. Then, the grand-mean average was computed within the same condition across all the participants to compare ERP components among the different conditions. The mean number of trials for each numerosity in each condition was 17.89/20.

Previous studies have found both parietal and temporo-occipital sites related with small and large numerosity modulations (Libertus et al., 2007; Hyde and Spelke, 2009; Liu et al., 2018). A similar scalp distribution is also commonly reported for an activity related to the maintenance of visual information in working memory (Pinal et al., 2014; Feldmann-Wüstefeld, 2021), especially in delayed match-to-sample tasks (McCollough et al., 2007; Ikkai et al., 2010; Pagano et al., 2014). Based on previous studies and visual inspection of the electrophysiological scalp topography, we focused our analyses on parietal-occipital regions. In order to describe the relatively integral brain activation for ERP components, electrodes P7, P5, P3, PO7, and PO3 on the left hemisphere, and electrodes P8, P6, P4, PO8, and PO4 on the right hemisphere were considered separately as the left and right regions of interest (ROIs).

After inspecting the ERP waveform evoked by the sample stimulus onset, we focused on the time window between sample stimulus offset and test stimulus onset in order to investigate the representation of numerosity during the delay period, when no visual stimulus appeared on the computer screen. However, complementary results regarding sample stimulus encoding during the first 300 ms can be found in Supplementary Figure 1. Waveforms for the 300–850-ms time window were extracted from the original epochs. In addition, to avoid possible long-lasting effects due to online processing of the sample numerosity, a new baseline correction was computed using the last 100 ms before stimulus offset (from 200 to 300 ms after sample stimulus onset). Such baseline correction has been commonly performed in match-to-sample paradigms (Barriga-Paulino et al., 2014, 2015; Pelegrina et al., 2020) in order to reduce the impact of stimulus encoding and highlight information maintenance during the delay period. A 50-ms long negative peak component was found after the stimulus offset at around 250 ms, followed by a 50-ms positive component. Hereafter, we refer to these components as D-N250 and D-P300 (with the letter D highlighting that these components were observed during the delay period). Global field power waveforms across all the conditions are provided in Supplementary Figure 2. The mean amplitude and peak latency of the D-N250 and D-P300 components, as well as the mean amplitude for a later time window between 320 and 550 ms in the two ROIs, were exported from Brainstorm for a preliminary three-way (numerosity, condition, and Hemisphere) repeated-measures ANOVA. Based on the preliminary results, the analysis was then conducted separately for each hemisphere.

In parallel with the behavioral investigation, we also evaluated the inflection point in neural components by fitting the bilinear model on individual mean amplitude as a function of the sample numerosity, separately for each ERP time window and condition. The mean bilinear model fit across time windows, condition, and hemisphere was *R*^2^_adj_ = 0.29. We then investigated potential differences between individual breaking points across the two conditions and compared the steepness of the slopes before and after the individual inflection point. Finally, we computed Spearman correlations to investigate a potential correspondence between behavioral and neural inflection point estimates.

## Results

### Behavioral Results

#### Performance in MTS Task

**FIGURE 2:**
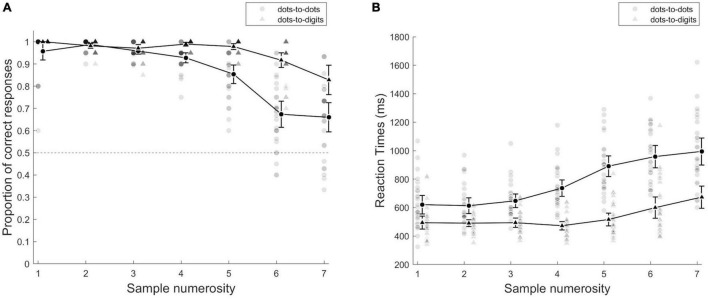
**(A)** Proportion of correct responses and **(B)** mean reaction times as a function of sample numerosity. Individual points represent single participants, and group averages are presented with black lines, separately for each condition. Error bars indicate 95% CI.

As a preliminary analysis of accuracy, we conducted a repeated measures ANOVA with condition (dots-to-dots and dots-to-digit) and sample numerosity (1–7) as within subject effects (see Figure 2A). This analysis revealed an overall higher accuracy in the dots-to-digit compared to dots-to-dots condition [*F*(1, 27) = 101.84, *p* < 0.001, η^2^*_*p*_* = 0.79], a significant effect of numerosity [*F*(2.36, 63.65) = 61.17, *p* < 0.001, η^2^*_*p*_* = 0.69], and a significant interaction [*F*(3.17, 85.48) = 18, *p* < 0.001, η^2^*_*p*_* = 0.4]. To better investigate performance in the two ranges, we then performed a repeated measures ANOVA with condition and range (based on group threshold) as within-subject effects, which confirmed the main effect of condition [*F*(1, 27) = 27.28, *p* < 0.001, η^2^*_*p*_* = 0.5] and revealed a significant effect of numerical range [*F*(1, 27) = 88.68, *p* < 0.001, η^2^*_*p*_* = 0.77], and a significant interaction between condition and range [*F*(1, 27) = 14.82, *p* < 0.001, η^2^*_*p*_* = 0.35]. The *post-hoc* tests showed an overall higher accuracy in small compared to large numerical range in both dots-to-dots [M(SD): M_small_ = 0.97 (0.02), M_large_ = 0.79 (0.09), *p* < 0.001, *d* = 1.87] and dots-to-digit [M_small_ = 0.98 (0.01), M_large_ = 0.87 (0.1), *p* < 0.001, *d* = 1.05] conditions. The difference between conditions, instead, emerged only in the large numerosity range (*p* < 0.001, *d* = 1.2).

We then considered individual thresholds, as the breakpoint individuated, by fitting a segmented model to each participant’s mean error rates as a function of numerosity. The comparison of the inflection points between conditions [*t*(26) = −3.41, *p* = 0.002, *d* = −0.66] revealed an overall smaller breakpoint for dots-to-dots [*M* = 3.96 (1.38)] compared to dots-to-digit [*M* = 5.1 (1.04)]. We then compared slopes of the pre-inflection and post-inflection segments of the bilinear model for the two conditions by two-way repeated measures ANOVA. The comparison of slopes indicated a difference between ranges [*F*(1, 26) = 39.59, *p* < 0.001, η^2^*_*p*_* = 0.6], with a steeper slope for the post-inflection segment [*M* = 0.12 (0.12)] compared to the pre-inflection line [*M* = −0.008 (0.08)]. The effect of condition and the two-way interaction was not significant.

The analyses on reaction times showed a similar pattern of results (see Figure 2B). The preliminary ANOVA with condition and sample numerosity as within-subject effects revealed a faster response in the dots-to-digit compared to the dots-to-dots condition [*F*(1, 27) = 114.8, *p* < 0.001, η^2^*_*p*_* = 0.81]. The main effect of numerosity [*F*(2.72, 73.46) = 102.44, *p* < 0.001, η^2^*_*p*_* = 0.79] and the interaction between numerosity and condition [*F*(1.74, 46.88) = 15.34, *p* < 0.001, η^2^*_*p*_* = 0.36] were also significant. The ANOVA comparing the two ranges similarly showed a significant effect of condition [*F*(1, 27) = 80.22, *p* < 0.001, η^2^*_*p*_* = 0.75] and numerical range [*F*(1, 27) = 174.61, *p* < 0.001, η^2^*_*p*_* = 0.87] and a significant interaction between the two factors [*F*(1, 27) = 10.26, *p* < 0.01, η^2^*_*p*_* = 0.27]. The *post-hoc* tests revealed faster performance in the small range than in the large numerical range in both the dots-to-dots [M_small_ = 629.17 (130.3), M_large_ = 872.03 (160.1), *p* < 0.001, *d* = −2.09] and dots-to-digit [M_small_ = 493.10 (78.03), M_large_ = 629.63 (191.54), *p* < 0.001, *d* = −1.17] conditions. Moreover, the participants were overall faster in the dots-to-digit compared to the dots-to-dots condition, both in the small (*p* < 0.001, *d* = −0.96) and large (*p* < 0.001, *d* = −1.7) ranges.

We then fitted individually a segmented model on mean reaction times as a function of numerosity with a fixed breakpoint based on individual subitizing thresholds estimated from error rates. A comparison of the pre- and post- inflection slopes showed a significant effect of range [*F*(1, 26) = 25.91, *p* < 0.001, η^2^*_*p*_* = 0.5], with a steeper positive slope in the post-inflection range [*M* = 96.82 (101.31)] compared to the pre-inflection one [*M* = 24.42 (73.16)]. The effect of the condition and the two-way interaction was not significant.

#### Correlations With LSC-SUA

We investigated the relationship between performance in the delayed match-to-sample task and arithmetic competence (see Table 1). A significant negative correlation was found between BIS in both the dots-to-dots and dots-to-digit conditions and MC timing. Notably, BIS in the dots-to-dots condition also showed a positive relationship with MC and AC scores.

**TABLE 1:** Descriptive statistics of scores in the two calculation subtests from LSC-SUA and correlation with balanced integration scores (BIS) from match-to-sample task.

				Pearson r	
		M	SD	BIS dots-to-dots	BIS dots-to-digits
LSC-SUA	MC scores (z)	0.10	0.94	0.40*	0.38
	MC total time (z)	–0.42	0.91	−0.51*	−0.46*
	AC scores (z)	0.37	0.91	0.43*	0.29

*p < 0.05 (FDR corrected with alpha = 0.05).

### Electrophysiological Results

**FIGURE 3:**
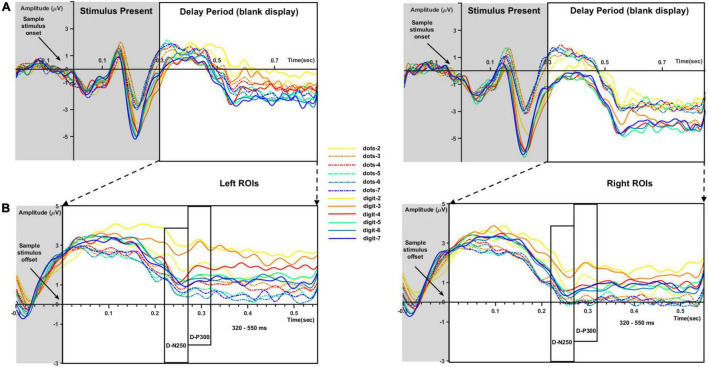
ERP waveforms for each sample numerosity (different colors) and condition (dots-to-dots and dots-to-digit with full and dashed lines, respectively) in left and right regions of interest (ROIs). **(A)** Waveforms evoked by sample numerosity onset. **(B)** Extracted time windows corresponding to the delay period (blank display). We considered for analysis the negative deflections around 250 ms after sample numerosity offset (D-N250), the positive deflections around 300 ms (D-P300), and the final slow waveforms between 320 and 550 ms.

Figure 3 represents the ERP waveforms evoked by sample numerosity onset (Figure 3A) and the averaged sub-epochs after sample stimulus offset (Figure 3B) on the left and right ROIs. Analyses of the components evoked by sample onset (i.e., P1 and N2) are reported in Supplementary Figure 1. For the analysis of the delay period, mean amplitude and peak latency for the D-N250 and D-P300 components and mean amplitude for the later 320–550-ms time window was inserted in separate three-way repeated-measure ANOVAs, with sample numerosity (7 levels: 2–7), condition (2 levels: dots-to-dots and dots-to-digit), and hemisphere (2 levels: left and right ROIs) as within-subject variables.

**FIGURE 4:**
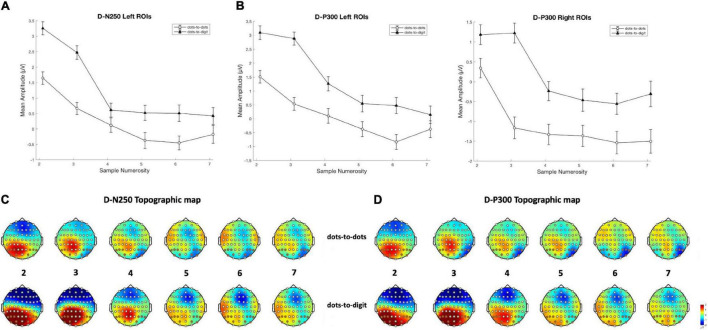
Mean amplitude results and topographical maps. Top row: mean amplitude for each sample numerosity in the two separate conditions for components **(A)** D-N250 on left ROIs and **(B)** D-P300 on both left and right ROIs. Error bars represent the standard error of the mean. Bottom row: topography for each sample numerosity in the two conditions for components **(C)** D-N250 and **(D)** D-P300. The maps were obtained from 220 to 270 ms for D-N250 and 270 to 320 ms for D-P300.

**FIGURE 5:**
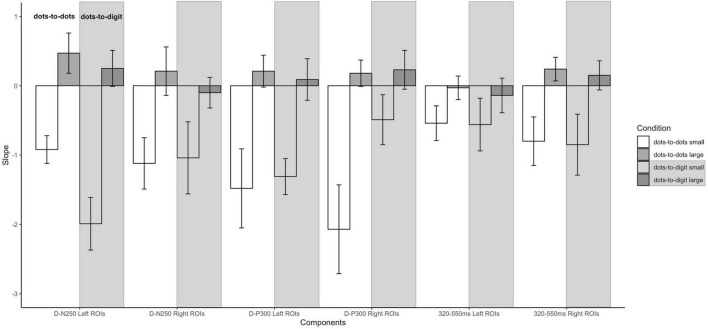
Slopes of pre- and post-inflection segments within each condition for each ERP component. Error bars represent the standard error of the mean.

As reported in Table 2, we found a significant effect of sample numerosity, condition, and hemisphere on mean amplitude for each of the time windows. The interaction between numerosity and condition was significant in the D-N250 and D-P300 components. Notably, the interaction between numerosity and hemisphere was significant in all the time windows; we, therefore, conducted separate repeated measures ANOVA for left and right ROIs. Since numerosity also interacted with the condition in all but one of the time windows, we retained the condition factor in these follow-up ANOVAs. We finally compared the slopes of mean amplitude as a function of numerosity in different ranges and conditions. The overall results of the latter analyses are shown in Table 3 and Figures 4, 5 and are presented below separately by component.

**TABLE 2:** Mean amplitude three-way repeated measures ANOVA results for numerosity, condition, and hemisphere effects on each time window.

		D-N250		D-P300		320–550 ms	
	*df*	*F*	*η_*p*_^2^*	*F*	*η_*p*_^2^*	*F*	*η_*p*_^2^*
Numerosity	5, 135	20.85***	0.44	19.04***	0.41	6.70***	0.20
Condition	1, 27	35.18***	0.57	48.44***	0.64	63.19***	0.70
Hemisphere	1, 27	13.87***	0.34	16.10***	0.37	7.19**	0.21
Numerosity × Condition	5, 135	2.65*	0.09	4.18**	0.13	1.35	0.05
Numerosity × Hemisphere	5, 135	3.81**	0.12	3.31**	0.11	3.58**	0.12
Condition × Hemisphere	1, 27	0.44	0.02	0.13	0.01	0.78	0.03
Numerosity × Condition × Hemisphere	5, 135	0.73	0.03	1.98	0.07	0.51	0.02

*p < 0.05, **p < 0.01, ***p < 0.001.

**TABLE 3:** Mean amplitude two-way repeated measures ANOVA results for numerosity and condition effects on left and right regions of interest (ROIs) separately for each time window.

		Left ROIs		Right ROIs	
	*df*	*F*	*η_*p*_^2^*	*F*	*η_*p*_^2^*
**D-N250**					
Numerosity	5, 135	23.44***	0.28	10.60***	0.15
Condition	1, 27	34.50***	0.09	21.57***	0.09
Numerosity × Condition	5, 135	2.69*	0.02	1.55	0.01
**D-P300**					
Numerosity	5, 135	18.46***	0.25	11.84***	0.16
Condition	1, 27	36.67***	0.12	36.69***	0.14
Numerosity × Condition	5, 135	3.79**	0.02	3.38**	0.03
**320–550 ms**					
Numerosity	5, 135	8.55***	0.14	3.53**	0.06
Condition	1, 27	59.47***	0.17	41.85***	0.16
Numerosity × Condition	5, 135	1.67	0.01	0.69	0.01

*p < 0.05, **p < 0.01, ***p < 0.001.

The three-way repeated measures ANOVA on peak latency for the D-N250 and D-P300 components did not yield significant main effects or interactions. Therefore, the peak latency data were not further analyzed.

#### D-N250 Component

For the D-N250 component, in the left ROIs, the ANOVA on mean amplitude revealed a significant interaction between condition and numerosity, and significant main effects (see Table 3). To better understand the effect of sample numerosity on the two conditions, a segmented model was then fitted on mean amplitude as a function of sample numerosity separately for the two conditions. No difference emerged between the inflection points in the dots-to-dots [M(SD) = 4.55 (1.06)] and dots-to-digit [M(SD) = 4.34 (1.07)] condition, and neither showed a significant correlation with behavioral breakpoints in the corresponding condition. Figure 5 represents the slopes of pre- and post-inflection segments within each condition. A two-way repeated measure ANOVA revealed a significant difference between pre- and post- inflection segments [*F*(1, 27) = 33.1, *p* < 0.001, η^2^*_*p*_* = 0.55], with a steeper negative slope for the pre-inflection line [M(SD) = −1.46 (1.54)] compared to the post-inflection one [M(SD) = 0.36 (1.44)]. A main effect of condition [*F*(1, 27) = 7.43, *p* = 0.01, η^2^*_*p*_* = 0.22] was also found because of an overall more negative slope for dots-to-digit [*M* = −0.87 (1.68)] compared to dots-to-dots [*M* = −0.23 (1.30)] condition. The interaction between condition and range was, however, not significant.

In the right ROIs, the ANOVA on mean amplitude revealed a significant main effect of condition and numerosity, whereas the interaction between the two was not significant. A higher mean amplitude was found in the dots-to-digit [M(SD) = 0.11 (2.36)] compared to dots-to-dots condition [M(SD) = −0.83 (2.44)]. However, in order to investigate the relationship between neural and behavioral inflection points, in this case, bilinear models were also estimated at the individual level separately for each condition. Breakpoints from individual segmented models were not significantly different in the two conditions [M(SD): dots-to-dots = 4.58 (1.37), dots-to-digits = 4.11 (1.21)], and no significant correlation was found between neural and behavioral breakpoints. Slope analysis revealed a significant effect of range [*F*(1, 27) = 7.35, *p* = 0.01, η^2^*_*p*_* = 0.21], with a steeper amplitude slope in pre-inflection [*M* = −1.08 (2.35)] compared to post-inflection [*M* = 0.06 (1.52)] segments. The effect of the condition and the interaction were not significant.

#### D-P300 Component

Considering the next positive peak component, the ANOVA on mean amplitude revealed significant two-way interactions and main effects of numerosity and condition on both left and right ROIs.

Breakpoints from the individual segmented model did not differ between the two conditions, both in the left [M(SD): dots-to-dots = 4.2 (1.16), dots-to-digit = 4.53 (1.16)], and in the right [M(SD): dots-to-dots = 4.27 (1.21), dots-to-digit = 4.39 (1.19)] ROIs, and no significant correlation was found between inflection points estimated from neural activity and behavioral performance.

Slope analysis for the left ROIs showed only a main effect of numerosity range [*F*(1, 27) = 16.41, *p* < 0.001, η^2^*_*p*_* = 0.38], with a steeper negative slope in the pre-inflection segment [*M* = −1.4 (2.19)] than in the post-inflection range [*M* = 0.15 (1.42)]. The effect of condition and the two-way interaction were not significant. Similarly, for the right ROIs, a main effect of range was found, with a steeper decreasing trend for pre-inflection [*M* = −1.28 (2.64)] than for post-inflection [*M* = 0.21 (1.25)] slopes [*F*(1, 27) = 18.07, *p* < 0.001, η^2^*_*p*_* = 0.4]. Moreover, in this hemisphere, we found a significant effect of condition, with a steeper overall decreasing trend for dots-to-dots [*M* = −0.95 (2.18)] compared to dots-to-digit [*M* = −0.13 (1.71)] condition [*F*(1, 27) = 5.5, *p* = 0.03, η^2^*_*p*_* = 0.17]. No significant interaction between condition and range was found.

#### The 320–550-ms Time Window

In this time window, the ANOVA on mean amplitude revealed in both hemispheres only significant main effects of numerosity and condition, with no two-way interactions. The dots-to-digit condition showed a greater mean amplitude than the dots-to-dots condition, both on the left [*M(SD)*: *M*_*digit*_ = 1.15 (2.5), *M_*dots*_* = −0.35 (2.51)] and right [*M*_*digit*_ = 0.17 (2.69), *M*_*dots*_ = −1.17 (2.9)] ROIs.

Similar with the previous two components, results from the individual segmented models did not reveal a significant difference between the breaking point of the two conditions, both in the left [M(SD): dots-to-dots = 4.47 (0.8), dots-to-digit = 4.14 (1.26)] and in the right [M(SD): dots-to-dots = 4.16 (0.96), dots-to-digit = 4.29 (1.28)] ROIs. Moreover, no correlation emerged between the inflection points estimated from mean amplitude and behavioral breakpoints in the corresponding condition.

For the slope comparison, no significant effect was found in the left hemisphere. However, in the right hemisphere, a significant range effect was found [*F*(1, 27) = 8.6, *p* = 0.007, η^2^*_*p*_* = 0.24] with a steeper decreasing trend for pre-inflection [*M* = −0.83 (2.09)] compared to post-inflection [*M* = 0.2 (1)] segments. The effect of condition and the interaction were not significant.

## Discussion

In this study, we investigated the encoding and maintenance of quantity information related to small and large numerosities in a delayed numerosity match-to-sample task. To this end, we analyzed electrophysiological activity between the presentation of a sample array of dots and a test numerical stimulus in non-symbolic or symbolic format. Importantly, the participants had to assess the number of objects in the sample stimulus because of the explicit comparison (same/different) requested after the test presentation (Sella et al., 2013). Through this manipulation, we aimed at individuating differences in the neural encoding of small and large numerosities, and to explore potential variations due to the use of cross-format matching. The numerical task allowed us to relate neural encoding and behavioral performance, as well as determine the functional relevance of our investigation, drawing a connection with real-world arithmetic abilities.

### Subitizing and Estimation

Task performance was modulated by numerical range. More specifically, both accuracy and reaction time results indicated better performance in the small range than in larger numerosities. In addition, the response pattern was different in the two ranges, as shown by the slope analysis. In the estimation range, accuracy showed a steady decrease, and reaction time showed a specular increase with numerosity. Conversely, compared to the estimation range, accuracy and reaction times remained relatively stable in the subitizing range. These results are in line with previous findings showing a differential pattern of response connected with small and large numerosities, compatible with the OTS vs. ANS distinction (Piazza, 2010; Choo and Franconeri, 2014). The estimated subitizing limit (between three and five items) fits well with existing literature on the capacity of the OTS in adults (Revkin et al., 2008; Burr et al., 2010), although some authors reported higher thresholds (Anobile et al., 2019). Moreover, the analysis on individual thresholds revealed a difference in the subitizing limit between the fully non-symbolic and mixed format conditions, in line with previous evidence showing that task settings can influence the subitizing span (e.g., Maldonado Moscoso et al., 2020). Despite our interpretation of the current findings as supporting the idea of a distinct mechanism underlying subitizing, we have previously mentioned that other accounts for this behavioral pattern have been proposed, postulating a single underlying mechanism (Sengupta et al., 2017; Cheyette and Piantadosi, 2020). However, our behavioral findings are complemented by electrophysiological results that clearly show modulation of parietal-occipital neural activity by numerical range.

The analyses of electrophysiological data focused on the time window between sample stimulus offset and test stimulus onset to investigate the representation of numerosity during the memory period before the comparison and response selection process. Note that the same logic has been widely used in neurophysiological studies investigating the coding of numerosity by single neurons during the delay period in a match-to-sample task (Nieder, 2005). In line with our initial hypotheses, in the memory retention interval after sample stimulus offset, we individuated two short time windows and one continuous slow wave sensitive to the number of dots in the array. Significant differences in amplitude between numerosities were found in a negative peak around 250 ms (D-N250) after stimulus offset, a subsequent positive peak (D-P300), and a later slow wave between 320 and 500 ms. More specifically, similar to previous results (Hyde and Spelke, 2012), we found a modulation of activity by numerical magnitude for small sets of items. Indeed, our analyses revealed a trend of increasing negativity for progressively larger sets of items (see Figure 4) up to numerosity 4, visible in all the time windows except for the 320–550-ms time window. Instead, we failed in finding a clear modulation by numerical magnitude in the estimation range, where neural activity presented consistently shallower slopes in comparison to the subitizing range. Although the investigated timing deviates from previous studies in number research, this result is generally in line with evidence differentiating the neural response to small and large numerosities (Hyde and Spelke, 2009).

These results are also similar to previous working memory studies showing that the magnitude of negative slow waves in the retention period increases, as a function of the number of elements to hold in memory, up to a capacity limit (McCollough et al., 2007). A possible interpretation would then relate our result as reflecting working memory processes involved in maintaining the representation of individual items in the sample, during the delay interval. Different from working memory research, however, the current task did not require tracking location, color, or other combinations of perceptual characteristics of the individual items, especially in the mixed-format condition where dot arrays had to be compared with Arabic numerals. Set size modulation on neural signatures of working memory in the context of an enumeration task has been previously reported by Pagano et al. (2014). More specifically, the authors presented an increase in CDA amplitude with numerosity changes in the subitizing range. The set-size modulation of neural activity in our numerical match-to-sample task could then suggest a similar implication of working memory processing even in the absence of an explicit need to track the information of single items. More specifically, in our results, the differential pattern shown by activity in relation to small and large numerosities supports the idea that individuation and working memory capacity would be involved in small exact numerical processing, in line with the distinction of the subitizing phenomenon from the intrinsic nature of the numerical magnitude representation of the ANS (Feigenson et al., 2004). Indeed, strikingly similar results emerge from working memory tasks involving the encoding of location or color (Marois and Todd, 2004) and enumeration tasks of small sets of objects (Cutini et al., 2014): in both cases, an increase of neural activity in the posterior parietal cortex with set size, leveling at approximately four elements, has been reported. However, even if behavioral studies have consistently related working memory capacity with subitizing limits (Piazza et al., 2011), we did not find a correspondence between behavioral subitizing capacity and neural activity, which also showed consistent threshold estimates across format conditions. This result is in line with previous research that failed in finding an association between set size CDA modulation and behavioral subitizing span, whereas a correlation has been found with an earlier component (N2pc) linked to spatial attention and object individuation, suggesting that other domain-general mechanisms may also play an important role in small numerosity processing (Pagano et al., 2014).

Alternatively, since the delayed match-to-sample task did not specifically target components associated with previous working memory studies, we must also consider the possibility that the present modulation of neural activity could be more directly related to number encoding. Comparative studies using single-cell recording showed that during a numerosity match-to-sample task, some neurons in the prefrontal and posterior parietal cortices of monkeys activate maximally in the delay period, following the sensory presentation of a specific number of items, both in small and large numerical ranges (Nieder and Merten, 2007). Other studies have revealed single neurons in the lateral intraparietal sulcus activating with a monotonic modulation as a function of numerosity (Roitman et al., 2007). These results leave room for speculation that the current modulation could be associated with the encoding of numerical information, especially through summation coding, rather than domain-general mechanisms. However, such an interpretation is difficult to reconcile with the differential modulation in the small and large ranges, especially since it has been demonstrated that monotonic modulation of neural activity in response to large numerosity is detectable from early processing stages (Park et al., 2016). Moreover, even though a monotonic change in neurophysiological activity has been reported for both small and large numerosities, even in early processing stages, differences between the two ranges in polarity, latency, and anatomical location of response suggest functional dissociation (Fornaciai and Park, 2017). In particular, while in the large range numerical modulation has been associated with approximate numerical coding, in the small range, it is thought to reflect the amount of attention allocated to individual objects (Hyde and Spelke, 2009). Still, our failure to find a clear modulation by magnitude in the estimation range could be attributed to differences in paradigm and numerical range, since the previous studies used passive viewing and larger sets of items compared to the range used in this study. Further research is then needed to better differentiate between domain-general and domain-specific effects. We suggest that the match-to-sample task could be an optimal ground for a similar investigation, offering insights into the retention of numerical information in relation to the first quantity, as well as into the process of numerical discrimination between the sample and test numerosities. Indeed, a previous investigation using a match-to-sample paradigm has found a modulation of neural activity in response to larger numerosities, but as a variation of amplitude in response to the second test stimulus, depending on the numerical distance from the sample set size (Paulsen and Neville, 2008; Paulsen et al., 2010).

### Behavioral and Neural Response to the Format Change

Analysis of the behavioral results in the two different conditions revealed higher performance in the cross-format presentation of sample and test stimuli, compared to the fully non-symbolic condition. After a non-symbolic sample, in the estimation range, the participants were significantly faster and more accurate when the test stimulus was a digit rather than a dot array, and a similar difference in speed also emerged in the subitizing range. These results are in direct contrast with evidence reporting a cognitive cost for the integration of symbolic and non-symbolic numerical information. Lyons et al. (2012) found that responses in a comparison task were slower and less accurate when adult participants had to compare an array of dots with a digit than when they had to judge two non-symbolic stimuli or two numerals, interpreting such effect as evidence of a dissociation between representations of symbolic and non-symbolic numerosities. Instead, the current results point toward facilitation in mixed-format compared to fully non-symbolic condition, coherent with the idea of a shared representation in which numerals differ from non-symbolic magnitudes in terms of sharpness of tuning. In this view, the finer tuning elicited from numbers would decrease uncertainty in the comparison, resulting in better and faster performance. Similar results were also reported by Marinova et al. (2020), who found that a cross-format presentation in a comparison task leads to an intermediate performance level between fully non-symbolic and fully symbolic conditions, respectively, associated with lower and higher accuracies. However, since they failed to find a ratio effect elicited by numerals, the authors concluded that different cognitive systems were implicated in the processing of symbolic and non-symbolic stimuli. Unfortunately, the lack of a fully symbolic condition in this study did not allow for the testing of a similar effect. Further investigations could better explore performance differences across formats, using a larger numerosity range or several ratios between a sample and test stimulus, to avoid the risk of ceiling effects. In addition, we must notice that the present facilitation in the mixed format could have been partially influenced by a fixed order of the dots-to-dots and dots-to-digit conditions, presented always in the first and second blocks, respectively.

At the neural level, the two conditions exhibited an overall similar pattern of result: in both the dots-to-dots and dots-to-digit conditions, ERP amplitudes were modulated by numerosity in all the examined time windows. In addition, even when a significant interaction between numerosity and condition emerged, such as in left D-N250 and bilateral D-P300, the two conditions revealed similar modulation between subitizing and estimation ranges. Since in both conditions the sensory stimulation before the delay period consisted of an array of dots, the similarity might appear to be a trivial result. On the other hand, the significant modulation of numerosity in the mixed-format condition, which required the comparison with a symbolic digit, seems more likely to reflect the encoding of the number of objects compared to the previous interpretations relating the amplitude change to the memory rehearsal of individual items. However, a possible reconciliation between the two hypotheses is offered by Pagano et al. (2014), who proposed that the involvement of working memory in numerical tasks could be connected to the necessity to maintain an active representation of individual items during mapping with a specific numerical value.

We hypothesized that cross-format presentation could induce a more precise encoding of sample numerosity. In line with this hypothesis, a difference in amplitude between the two conditions emerged during the entire delay period, with responses to dots-to-digit trials eliciting an overall more positive activity compared to the dots-to-dots condition. Importantly, given the non-symbolic nature of the sample stimulus in both conditions, a difference in amplitude under numerical modulation between the two blocks could be attributed to the task-relevant information held in memory rather than potential discrepancies in sensory stimulation. This phenomenon is acknowledged in working memory studies, where differences in neurophysiological activity have been reported in response to identical stimuli, depending on features that participants were required to focus on (Woodman and Vogel, 2008). Moreover, we found a significant difference in slope present in the left hemisphere on D-N250, where the amplitude in the dots-to-digit condition showed a steeper decrease compared to fully non-symbolic trials. Instead, contrary to our hypothesis, we found the opposite effect in the right ROIs on the later D-P300 component, where the amplitude showed a stronger decrease in the dots-to-dots condition compared to the mixed format block. This result is consistent with a top-down modulation on the encoding of numerical magnitude due to specific task settings. A larger spacing of neural activity between adjacent numerosities could be the expression of an enhancement of the functional coding of the numerical information, emerging at different time points in left parietal sites in preparation of a comparison with an exact numeral and in the right hemisphere before non-symbolic targets. Converging evidence suggests that while bilateral regions near IPS have been associated with non-symbolic numerical processing, left-lateralized frontoparietal circuits could be related to the processing of exact numerical information, possibly related to the involvement of linguistic mechanisms in symbolic numerical processing (Ansari, 2007; Piazza et al., 2007). However, these results must be interpreted with caution, as it must be noted that evidence in favor of hemispheric specialization emerged only in analyses on individual slopes.

Despite the pre-delay baseline correction, we cannot exclude that the effects on the delay period might be influenced by the initial processing of the visual stimuli. In particular, the difference in amplitude between the two conditions could be ascribed to the effect of attentional processes involved in the initial processing phase and persisted in the memory period. This is consistent with a specular modulation of N2 (see Figure 2 and Supplementary Figure 1). On one hand, the increased attentional engagement in the dots-to-digit condition might be related to task difficulty (Dong et al., 2015). However, while format condition has been shown to impact behavioral performance mainly in the estimation range, at the neural level, this effect was consistent for small and large numerosities. Moreover, behavioral results indicate better performance in the dots-to-digit compared to the dots-to-dots condition. On the other hand, attentional differences could also be more specifically attributed to the involvement of different individuation mechanisms in the two blocks, such as groupitizing strategies (Starkey and McCandliss, 2014), which have been shown to rely on attentional resources (Maldonado Moscoso et al., 2020). Indeed, the dots-to-digit condition, requiring higher enumeration precision, could encourage participants to cluster the elements of the arrays to facilitate enumeration. This interpretation is also consistent with our behavioral results, showing better performance and a higher subitizing threshold in the dots-to-digit condition (also see Maldonado Moscoso et al., 2020; Anobile et al., 2021). However, we must note that the sample stimulus was present on screen for only 300 ms, discouraging exact enumeration in both conditions and that the location of the dots in the array was randomly selected to avoid consistent grouping. We believe that future investigations specifically addressing this hypothesis could benefit from information regarding gaze movement during the initial processing phase (Schindler et al., 2020).

Finally, we must also acknowledge the possibility of confounding effects deriving from a blocked procedure. In particular, we cannot exclude that the overall difference in amplitude between the two conditions, both in the early time window and, as a sustained effect, the delay period, could be partially associated with reduced neural activity in the dots-to-dots condition connected with repetition suppression effects due to the uniform presentation of dot arrays (Summerfield and de Lange, 2014).

### Basic Numerical Abilities and Mathematical Skills

The correlational analysis revealed a significant link between performance in the match-to-sample task and math abilities. More specifically, performance in the dots-to-digit trials was related to the speed in the mental calculation test, and performance in both dots-to-dots was also related to the mental calculation scores. In addition, performance in the dots-to-dots condition correlated with approximate computation abilities. The significant relationship between speed of arithmetic computation and overall performance in the mixed-format condition is in line with previous evidence reporting a reliable link between math scores and symbolic comparison (Schneider et al., 2017). A similar connection with performance in fully non-symbolic presentation is particularly relevant for the ongoing debate on the connection between magnitude processing and broader mathematical skills. Recently, it has been proposed that non-symbolic numerical representation and symbolic math abilities would present a stronger link during the first steps of arithmetic knowledge acquisition, progressively differentiating because of increasing experience with formal mathematics (Ansari, 2008). This interpretation is supported by contradicting results on the relationship between mathematical skills and numerical acuity in non-symbolic comparison or estimation tasks (Castronovo and Göbel, 2012; Price et al., 2012; Szkudlarek et al., 2021). In contrast, the current result highlighted a relationship between magnitude processing and arithmetic computation in educated adults, suggesting a more continuous link during the life span. One possible explanation of the current result lies in the nature of the measures used. Previous studies have, revealed that non-symbolic processing could be differentially related to different aspects of math abilities (Lourenco et al., 2012). In particular, arithmetic computations that cannot be solved *via* rote memory, such as in the LSC tests administered in this investigation, are thought to rely more on magnitude processing and show a stronger relationship with precision in non-symbolic tasks (Piazza et al., 2010). Similar reasoning applies to the a match-to-sample paradigm as a measure of basic numerosity processing, which could partially diverge from acuity measures more commonly derived from comparison and estimation tasks. Notably, previous associations between performance in a similar computerized task and several tests of numerical competence have been reported in typically developing children and children with Down syndrome (Sella et al., 2013, 2021).

The nature of the relationship between numerosity processing and math skills is still a matter of debate since evidence of bidirectional influence between the precision of magnitude representation and math knowledge has been found (Elliott et al., 2019). One view proposes that magnitude processing might scaffold the acquisition of symbolic numerals, with an impact on broader symbolic math abilities (Libertus et al., 2013). In this view, even after formal learning, approximate number representation could provide support to basic operations, helping in the intuitive detection of errors (Feigenson et al., 2013). This interpretation is also supported by the current results, in particular by the significant correlation emerging between performance in non-symbolic trials and scores in the approximate computation test, where the participants had to quickly individuate a correct response by means of comparing the order of magnitudes of the multiple choices provided. However, even if our results are in support of a link between non-symbolic and symbolic representations and arithmetic abilities in adults, precise conclusions on the direction of this relationship cannot be drawn from the current correlational analysis.

## Conclusion

To conclude, our results provide new evidence for a functional dissociation between subitizing and estimation mechanisms. During an explicit numerical match-to-sample task, in addition to the behavioral signatures of OTS and ANS, we found that the two ranges were characterized by differential modulation of parietal-occipital neural activity by numerical information. Using ERPs, we demonstrated clear similarities with working memory neural signatures during the retention or encoding period of small numerical quantities, suggesting an implication of domain-general mechanism in small number processing. We also report evidence of top-down modulation of neural processing based on the demands of the task. Differences in ERP amplitude and numerical modulation suggest a qualitative variation in the encoding of numerical information in response to identical stimuli as a function of single vs. mixed-format comparison. The relevance of the current results is further emphasized by the significant relationship between performance in the delayed numerosity match-to-sample task and real-life mathematical skills, thereby supporting its validity for assessing basic number processing skills.

## Data Availability Statement

The raw data supporting the conclusions of this article will be made available by the authors, without undue reservation.

## Ethics Statement

The studies involving human participants were reviewed and approved by the Psychological Science Ethics Committee of the University of Padova. The patients/participants provided their written informed consent to participate in this study.

## Author Contributions

WF, GD, CS, and MZ contributed to the conception and design of the study. WF and GD performed the data collection. WF, SD, CS, and MZ contributed to the analysis and interpretation of data. WF and SD wrote the first draft of the manuscript. All the authors contributed to manuscript revision and approved the submitted version of the manuscript.

## Conflict of Interest

The authors declare that the research was conducted in the absence of any commercial or financial relationships that could be construed as a potential conflict of interest.

## Publisher’s Note

All claims expressed in this article are solely those of the authors and do not necessarily represent those of their affiliated organizations, or those of the publisher, the editors and the reviewers. Any product that may be evaluated in this article, or claim that may be made by its manufacturer, is not guaranteed or endorsed by the publisher.
