# Supplementary material for: Electrophysiological Signatures of Numerosity Encoding in a Delayed Match-to-Sample Task
Source: Front Hum Neurosci. 2022 Jan 4;15:750582. doi: 10.3389/fnhum.2021.750582 (PMC8764258; doi:10.3389/fnhum.2021.750582)
Supplement: Supplementary file 1 [file Data_Sheet_1.docx]

Supplementary Material

**Early time window waveform after sample stimulus onset**

We investigated the first 300ms time windows, focusing on P1 (130-185 ms) and N2 (185-270 ms) components (see Figure S1). Mean amplitude for P1 and N2 components were inserted in separate three-way repeated-measure ANOVAs, with sample numerosity (7 levels: 2 to 7), condition (2 levels: dots-to-dots and dots-to-digit) and hemisphere (2 levels: left and right ROIs) as within-subject factors. For P1 component, the main effects of condition and hemisphere were significant (*F*(1, 27) = 6.16, *p* = .02, *η²_p_* = 0.19; *F*(1, 27) = 5.70, *p* = .02, *η²_p_* = 0.17). The two-way interactions between condition and hemisphere (*F*(1, 27) = 19.26, *p* < .001, *η²_p_* = 0.42) as well as between numerosity and condition (*F*(5, 135) = 2.39, *p* = .04, *η²_p_* = 0.08) were significant. For N2 component, the condition main effect and the interaction between condition and hemisphere were significant, (*F*(1, 27) = 56.38, *p* = < .001, *η²_p_* = 0.68; *F*(1, 27) = 18.76, *p* < .001, *η²_p_* = 0.41, respectively). No other effect was significant.


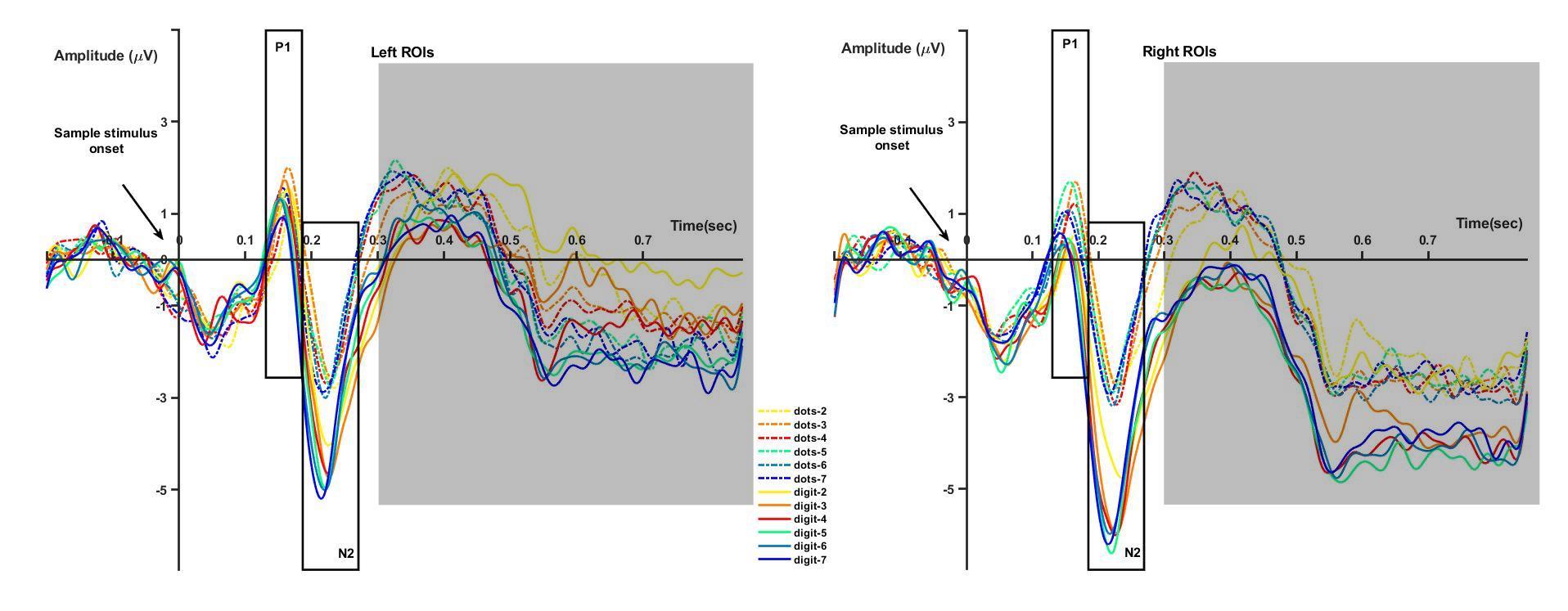


**Figure S1.** The waveform evoked by sample stimulus onset (early time windows).


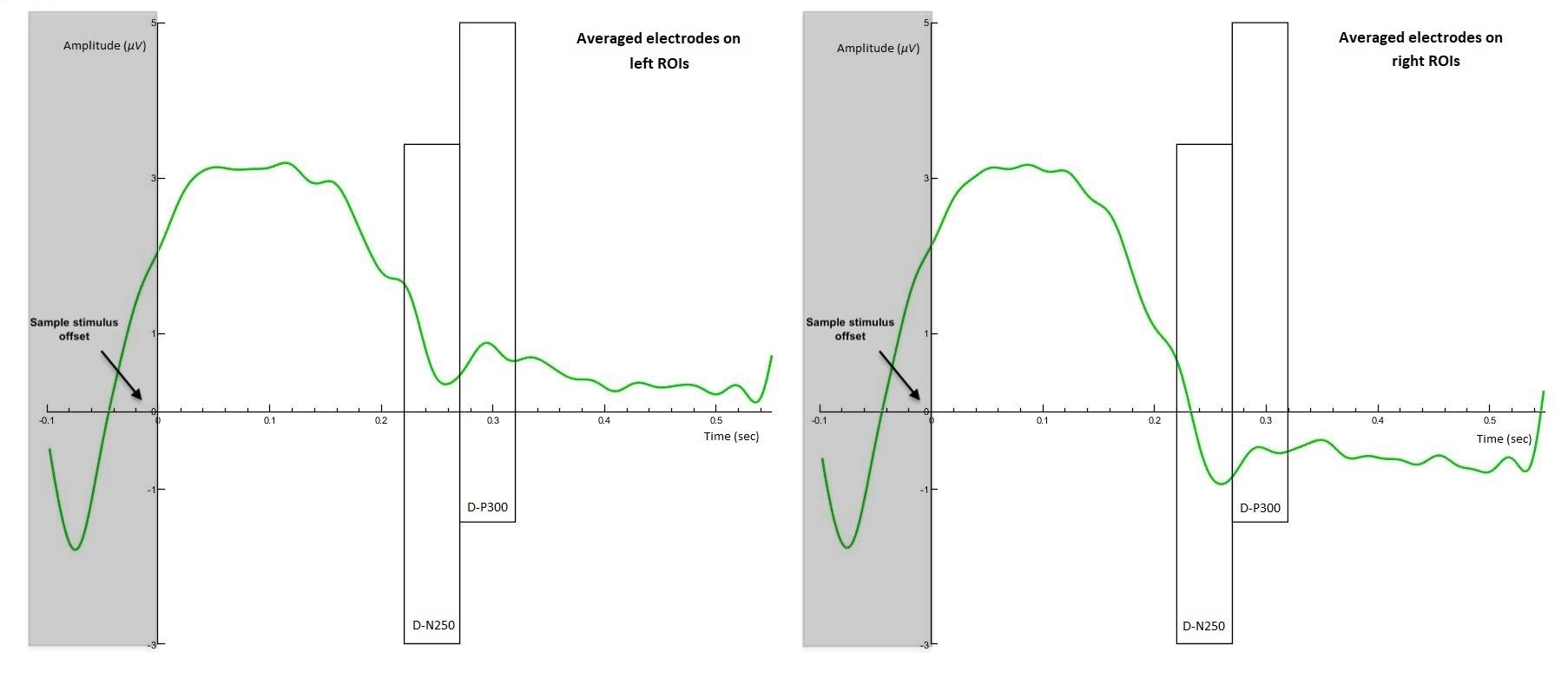
 **Figure S2.** Global Field Power waveforms across sites and conditions for the two ROIs after stimulus offset. Rectangular areas highlight the selected time windows named as D-N250 (220-270 ms) and D-P300 (270-320 ms).
